# Case Report: rapid clinical complete response to Iparomlimab and Tuvonralimab combined with radiotherapy and chemotherapy in a patient with MSS/pMMR locally advanced rectal cancer

**DOI:** 10.3389/fonc.2026.1936564

**Published:** 2026-09-15

**Authors:** Jue Wang, Tao Yang, Jingrui Zhou, Weimin Chen, Guiwei Wang, Chaoyu Mei, Ziwei Chen, Jibiao Li, Tao Shen

**Affiliations:** 1 Department of Colorectal Surgery, The Third Affiliated Hospital of Kunming Medical University, Kunming, Yunnan, China; 2 Kunming Medical University, Kunming, Yunnan, China; 3 People's Hospital of Nujiang Lisu Autonomous Prefecture, Lushui, Yunnan, China; 4 Zhumadian Central Hospital, Zhumadian, Henan, China

**Keywords:** combined immunotherapy, immune checkpoint inhibitors, Iparomlimab and Tuvonralimab, MSS/pMMR rectal cancer, PD-1/CTLA-4 bispecific antibody

## Abstract

**Objective:**

To investigate the efficacy and safety of Iparomlimab and Tuvonralimab, a PD-1/CTLA-4 bispecific antibody, in combination with chemoradiotherapy for a patient with microsatellite stable/mismatch repair proficient (MSS/pMMR) locally advanced rectal cancer (LARC), and to analyze the mechanism of achieving short-term clinical complete response (cCR).

**Methods:**

This case report presents a 61-year-old male with stage cT3N2aM0 IIIB MSS/pMMR rectal cancer. The patient received neoadjuvant therapy with Iparomlimab and Tuvonralimab (300mg per dose) plus XELOX regimen chemotherapy and radiotherapy (45Gy/25F). Responses were regularly assessed via digital rectal examination, imaging, endoscopy, and pathology.

**Results:**

After treatment, clinical complete response (cCR) was initially evidenced by MRI at week 12 and subsequently confirmed by endoscopic assessment combined with pathologic biopsy at week 20. No grade 3 or higher immune-related adverse events occurred. At the last follow-up in March 2026, no recurrence was observed.

**Conclusion:**

This case suggests that Iparomlimab and Tuvonralimab combined with chemoradiotherapy may induce rapid cCR in MSS/pMMR LARC, offering a promising organ-preserving strategy through immunotherapy. Further large-sample studies are warranted.

## 1 Introduction

Colorectal cancer (CRC) is a highly prevalent malignant tumor worldwide. According to the GLOBOCAN 2022 data, it ranks the third in terms of incidence rate and the second in mortality rate (1). In China, the disease burden of CRC remains heavy, with over 500,000 new cases reported in 2022, among which approximately 20% were diagnosed as locally advanced rectal cancer (LARC) or metastatic stage at initial presentation (2). Rectal cancer accounts for 30%–40% of all CRC cases. The traditional treatment paradigm involves neoadjuvant chemoradiotherapy followed by interval chemotherapy (when applicable) and subsequent surgery. However, this regimen has drawbacks including a prolonged treatment cycle of 6–8 months, severe toxic and side effects, low anus-preservation rate, and high risk of distant metastasis (3, 4). Immune checkpoint inhibitors (ICIs) have achieved breakthroughs in the treatment of microsatellite instability-high (MSI-H) rectal cancer. Nevertheless, more than 95% of cases belong to the microsatellite-stable (MSS)/proficient mismatch repair (pMMR) subtype, which responds poorly to single-agent ICIs due to the immunosuppressive tumor microenvironment (so-called “cold tumors”) (5, 6). The underlying mechanism lies in the low tumor mutational burden and sparse T-cell infiltration of such tumors, which hinder the effective activation of anti-tumor immune responses (7, 8).

Given the aforementioned predicaments, to explore more effective therapeutic regimens for MSS rectal cancer, immune combination therapy has emerged as one of the key strategies to overcome the challenges in immunotherapy for this subtype. By activating tumor-associated antigen-specific immune cells and enhancing T-cell infiltration in the tumor microenvironment, immune combination therapy can convert “immunologically cold tumors” into “immunologically hot tumors”.

The evolution of tumor immunotherapy has demonstrated that PD-1 and CTLA-4, as key immune checkpoint targets, regulate T-cell function through distinct mechanisms. They exhibit a synergistic effect in T-cell activation: CTLA-4 primarily modulates the early activation and tumor infiltration of T cells (9), while PD-1 acts on the functional suppression of T cells within the tumor microenvironment. Dual-target blockade can simultaneously enhance T-cell mobilization, tumor infiltration, and cytotoxicity (10). In the exploration of immune combination therapy, Iparomlimab and Tuvonralimab (Qibeian) — the first domestically developed dual-functional combined antibody targeting both PD-1 and CTLA-4 in China — has demonstrated unique innovative value. Its advent provides new options and insights for immune combination therapy, holding promise to further improve the treatment efficacy and survival benefits of patients with MSS rectal cancer. However, whether Iparomlimab and Tuvonralimab therapy, as a form of immune combination therapy, can yield superior outcomes and favorable safety profiles in pMMR/MSS rectal cancer still awaits further investigation and research.

This paper reports a case of a patient with MSS/pMMR rectal cancer who achieved a rapid clinical complete response (cCR) after undergoing neoadjuvant therapy consisting of Iparomlimab and Tuvonralimab combined with chemoradiotherapy, and during the follow-up period of another year, no recurrence was observed. Thereby providing a reference for future studies.

## 2 Case report

### 2.1 Description

A 61-year-old male patient was admitted to our hospital due to “altered bowel habits accompanied by hematochezia in October 2024”. Initial colonoscopy and pathological examination at another hospital confirmed a diagnosis of rectal adenocarcinoma. No relevant special treatment was conducted before coming to our hospital for treatment. The patient had a history of good health, with a 30-year smoking history (5 cigarettes per day) and a 30-year drinking history (50 g of alcohol per day). The patient denies any past medical history, allergy history, family history of infectious diseases, genetic diseases, or history of tumors. Physiological assessment indicated overweight status (BMI 26.83 kg/m²), good performance status (ECOG-PS score 1, KPS score 100), and nutritional risk (NRS 2002 score 3). A re-examination at our hospital confirmed the preliminary diagnosis of rectal malignant tumor.

### 2.2 Timeline

(**Table 1**).

**Table 1:** Timeline of key events from presentation to last follow-up.

Date	Event/intervention	Findings/outcome	Category
10/2024	Onset of symptoms, Presented to the local hospital	Altered bowel habits, hematochezia	Clinical
10/2024	Colonoscopy (local hospital)	3×4 cm mass in distal rectum	Diagnostic
11/2024	Pathology (local hospital)	Adenocarcinoma	Diagnostic
11/2024	Pathology review & IHC (our hospital)	High-grade intraepithelial neoplasia with focal intramucosal adenocarcinoma; pMMR confirmed	Diagnostic
11/2024	Staging MRI/CT	cT3N2aM0, stage IIIB	Diagnostic
11/2024 –02/2025	Immunotherapy	4 cycles of Iparomlimab and Tuvonralimab	Treatment
11/2024 – 02/2025	Chemotherapy	3 cycles of XELOX	Treatment
12/2024 – 01/2025	Radiotherapy	45 Gy/25F	Treatment
02/2025	MRI	cT0N0, mrTRG 1, no significant high signal on DWI sequence, cCR confirmed by MRI	Follow-up
02/2025 – 04/2025	Immunotherapy	5 cycles of Iparomlimab and Tuvonralimab	Treatment
02/2025 – 04/2025	Chemotherapy	4 cycles of XELOX	Treatment
04/2025	CT	Marked tumor regression	Follow-up
04/2025	Colonoscopy + biopsy	White scar, negative biopsy, cCR confirmed by colonoscopy + biopsy	Follow-up
07/2025	CT	Stable disease; no new lesions	Follow-up
07/2025	MRI	mrTRG 1	Follow-up
10/2025	CT and MRI	mrTRG 1, No progression compared to prior	Follow-up
03/2026	CT and MRI	mrTRG 1 (no residual tumor)	Follow-up
03/2026	Colonoscopy + biopsy	No recurrence, stable scar	Follow-up

### 2.3 Diagnostic assessment

Digital rectal examination: A mass was palpable approximately 3–4 cm from the anal verge, located in the 3 to 6 o’clock position, with luminal narrowing. The mass was firm, poorly mobile, and irregular, with no tenderness. No blood staining was observed on the withdrawn finger.

Colonoscopy (another hospital, October 2024): An irregular 3×4 cm mass was detected in the distal rectum, characterized by friability and easy bleeding. Endoscopic diagnosis: rectal mass.

Pathology (another hospital, November 2024): (Rectal) adenocarcinoma.

Pathological review (our hospital, November 2024): High-grade intraepithelial neoplasia of the rectum, with focal intramucosal adenocarcinoma. Immunohistochemistry results: proficient mismatch repair (pMMR) (**Figure 1A**).

**Figure 1:**
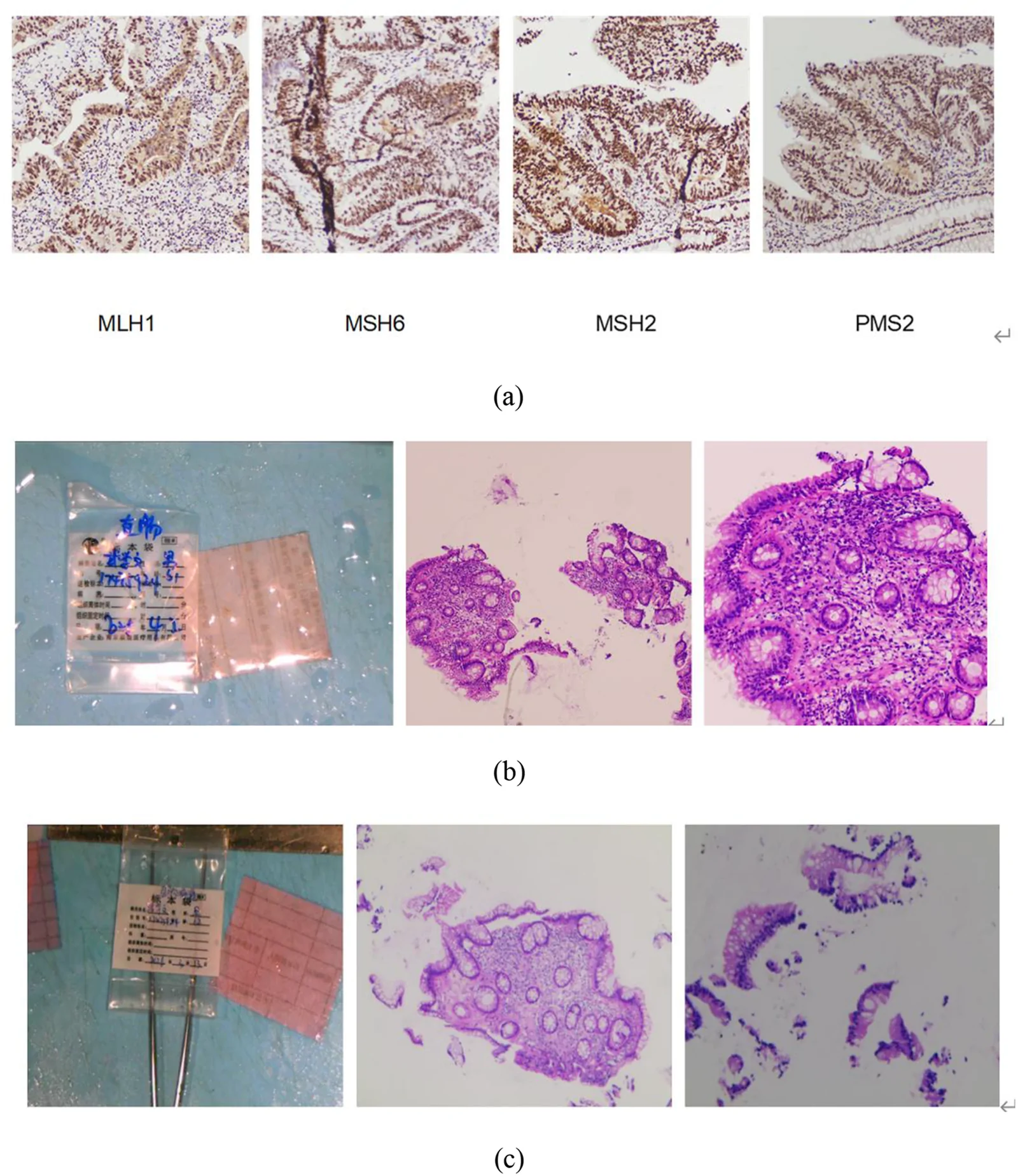
Immunohistochemistry at initial diagnosis and two pathological examinations during follow-up. **(A)** Immunohistochemistry results on November 2024, pMMR; **(B)** Pathological examination on April 2025; **(C)** Pathological examination on March 2026.

CT scan (November 2024): Irregular thickening of the right wall of the middle and distal rectum, with a maximum thickness of approximately 1.5 cm, showing significant heterogeneous enhancement after contrast administration. The boundary between the lesion and the right levator ani muscle was poorly defined. Multiple perienteric lymph nodes were visualized, with the largest one measuring approximately 0.3 cm in short diameter. Diagnostic opinion: thickening of the right wall of the middle and distal rectum, accompanied by perienteric lymph node visualization (**Figure 2A**).

**Figure 2:**
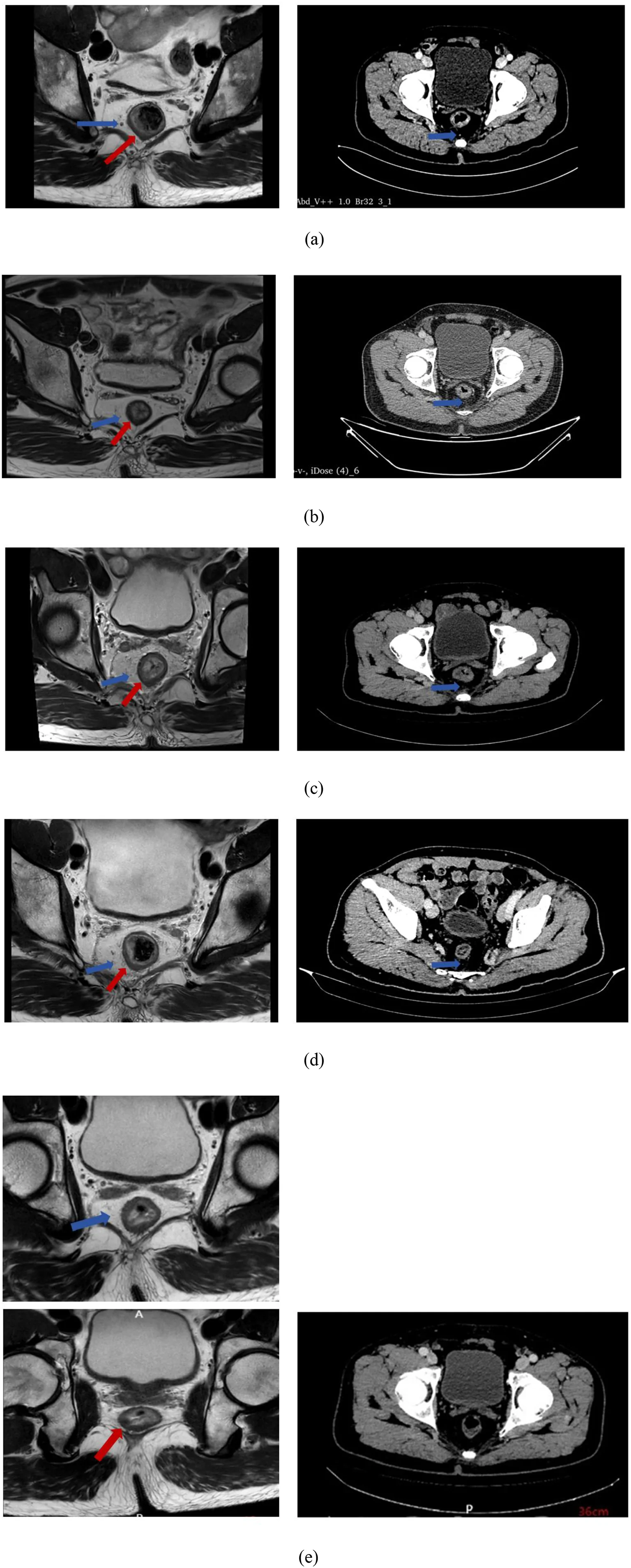
Serial imaging examinations performed at initial diagnosis and during the follow-up period, comprising a total of five time points. The left and right panels show the MRI and CT images, respectively. MRI showing the tumor invasion depth (red arrow) and enlarged lymph nodes (blue arrow). CT showing the perienteric enlarged lymph node (blue arrow). **(A)** Baseline imaging examinations on November 2024. MRI: The lower edge of the rectal mass was approximately 4.3 cm from the anus. The tumor invaded the muscularis propria but did not penetrate the serosa. The length of the lesion segment was about 3.4 cm, involving the entire circumference of the intestinal lumen. Multiple lymph nodes were detected around the superior rectal artery and within the mesorectal fascia (approximately 4 nodes), with the largest one measuring 6 mm × 4 mm. CT: Irregular thickening of the right wall of the middle and distal rectum, with a maximum thickness of approximately 1.5 cm, showing significant heterogeneous enhancement after contrast administration. The boundary between the lesion and the right levator ani muscle was poorly defined. Multiple perienteric lymph nodes were visualized, with the largest one measuring approximately 0.3 cm in short diameter. **(B)** MRI on February 2025 and CT on April 2025. MRI: Mild thickening and edema of the intestinal wall at the original rectal tumor site, prominent at the mucosal surface. The lesion was located below the peritoneal reflection. Anal complex **(A)**: no involvement. T stage: T0 (no obvious mass at the original lesion site, no significant high signal on DWI sequence). Post-neoadjuvant therapy MRI tumor regression grade (TRG): mrTRG 1 (no residual tumor); increased tumor mucin component: no. Diagnostic opinion: Post-neoadjuvant therapy changes of middle and distal rectal cancer (cT0N0), rectal edema. Anal canal was not involved. MRF (-). EMVI (-). Multiple lymph nodes visualized around the superior rectal artery and within the mesorectal fascia, with the short diameter of approximately 0.3 cm, uniform signal, uniform enhancement. CT: Uneven thickening of the middle and distal rectal wall, with maximum thickness of approximately 1.3 cm, showing significant heterogeneous enhancement after contrast. Multiple perienteric lymph nodes visualized, with the largest short diameter of approximately 0.2 cm. **(C)** MRI on July 2025 and CT on July 2025. MRI: Post-neoadjuvant therapy MRI tumor regression grade (TRG): mrTRGI (no residual tumor). T stage: T0 (no obvious mass at the original lesion site, no significant high signal on DWI sequence). Lymph nodes around the superior rectal artery and within the mesorectal fascia were basically unchanged; no abnormally enlarged lymph nodes in the lower retroperitoneum and bilateral iliac vessels of the pelvic cavity. Anal canal was not involved. MRF (-). EMVI (-). CT: Thickening of the middle and distal rectum with perienteric lymph nodes; mild thickening of pelvic mesentery and fascia; no significant changes compared with prior scan. Multiple perienteric lymph nodes visualized, largest with short diameter of approximately 0.2 cm, well-defined borders, and relatively uniform enhancement after contrast. **(D)** MRI and CT on October 2025. mrTRG1: No residual tumor, reduced rectal edema compared to before. T stage:T0, No obvious mass was found in the original lesion area, and no significant high signal was observed in the DWI sequence. N stage: Lymph nodes around the superior rectal artery and within the mesorectal fascia are displayed, with a short diameter of about 0.3cm, uniform signal, and uniform enhancement after enhancement. CT: Uneven thickening of the middle and lower rectal walls, with a maximum thickness of about 1.3cm, and uneven enhancement. Multiple lymph nodes around the intestine are shown, with larger ones having a short diameter of about 0.2cm, clear boundaries, and moderate enhancement and strengthening. **(E)** MRI and CT on March 2026. The small lymph nodes previously noted are no longer definitively visualized on the CT. The rest is roughly the same as before.

MRI (November 2024): The lower edge of the rectal mass was approximately 4.3 cm from the anus. The tumor invaded the muscularis propria but did not penetrate the serosa. The length of the lesion segment was about 3.4 cm, involving the entire circumference of the intestinal lumen. Multiple lymph nodes were detected around the superior rectal artery and within the mesorectal fascia (approximately 4 nodes), with the largest one measuring 6 mm × 4 mm. Diagnostic opinion: middle and distal rectal cancer (**Figure 2A**).

Final Diagnosis (No diagnostic challenges): Rectal adenocarcinoma, clinical stage cT3N2aM0 IIIB (MSS/pMMR subtype), the final clinical stage was determined as cT3N2aM0 IIIB based on comprehensive MRI findings.

### 2.4 Details on the therapeutic intervention

- Immunotherapy: Iparomlimab and Tuvonralimab 300 mg, intravenous drip (6 vials per administration, 50 mg per vial), once every 3 weeks was administered on a compassionate-use basis, as this PD-1/CTLA-4 bispecific antibody is not yet approved for neoadjuvant treatment of locally advanced rectal cancer. Written informed consent was obtained after comprehensive discussion of the off-label nature of the regimen, potential benefits, and risks, for 9 cycles (November 2024 to April 2025).
- Chemotherapy: XELOX regimen (Oxaliplatin 130 mg/m² on day 1 + Capecitabine 1000 mg/m² twice daily on days 1–14), for 7 cycles (November 2024 to April 2025). This intensified regimen was selected by multidisciplinary team (MDT) consensus, considering the patient’s relatively young age, good performance status (ECOG-PS 1), and the patient had a pronounced desire for maximal tumor downstaging to optimize the probability of sphincter preservation.
- Radiotherapy: A total of 25 fractions of radiotherapy (45 Gy/25F) were administered from December 2024 to January 2025.

### 2.5 Follow-up and outcomes

Treatment response was evaluated through nearly one year of follow up after completion of the therapy.

Digital rectal examination: No masses were palpated upon insertion up to 7 cm, and no blood staining was noted on the withdrawn finger.

On February 2025, MRI demonstrated mild thickening and edema at the primary tumor site with mrTRG 1 (no residual tumor), T stage: T0 (no obvious mass at the original lesion site, no significant high signal on DWI sequence). Along with reduced perirectal lymph nodes (short diameter ~0.3 cm) compared with baseline, uniform signal, uniform enhancement. Diagnostic opinion: Post-neoadjuvant therapy changes of middle and distal rectal cancer (cT0N0) (**Figure 2B**). On April 2025, colonoscopy revealed a white linear constrictive scar 4 cm from the anal verge, with clear capillary network and glandular openings under NBI (endoscopic cCR suspected) (**Figure 3A**), and pathological biopsy yielded only a small amount of mucosal tissue (**Figure 1B**). Concurrent CT showed mildly decreased wall thickening and smaller perienteric lymph nodes (largest short diameter ~0.2 cm) (**Figure 2B**).

**Figure 3:**
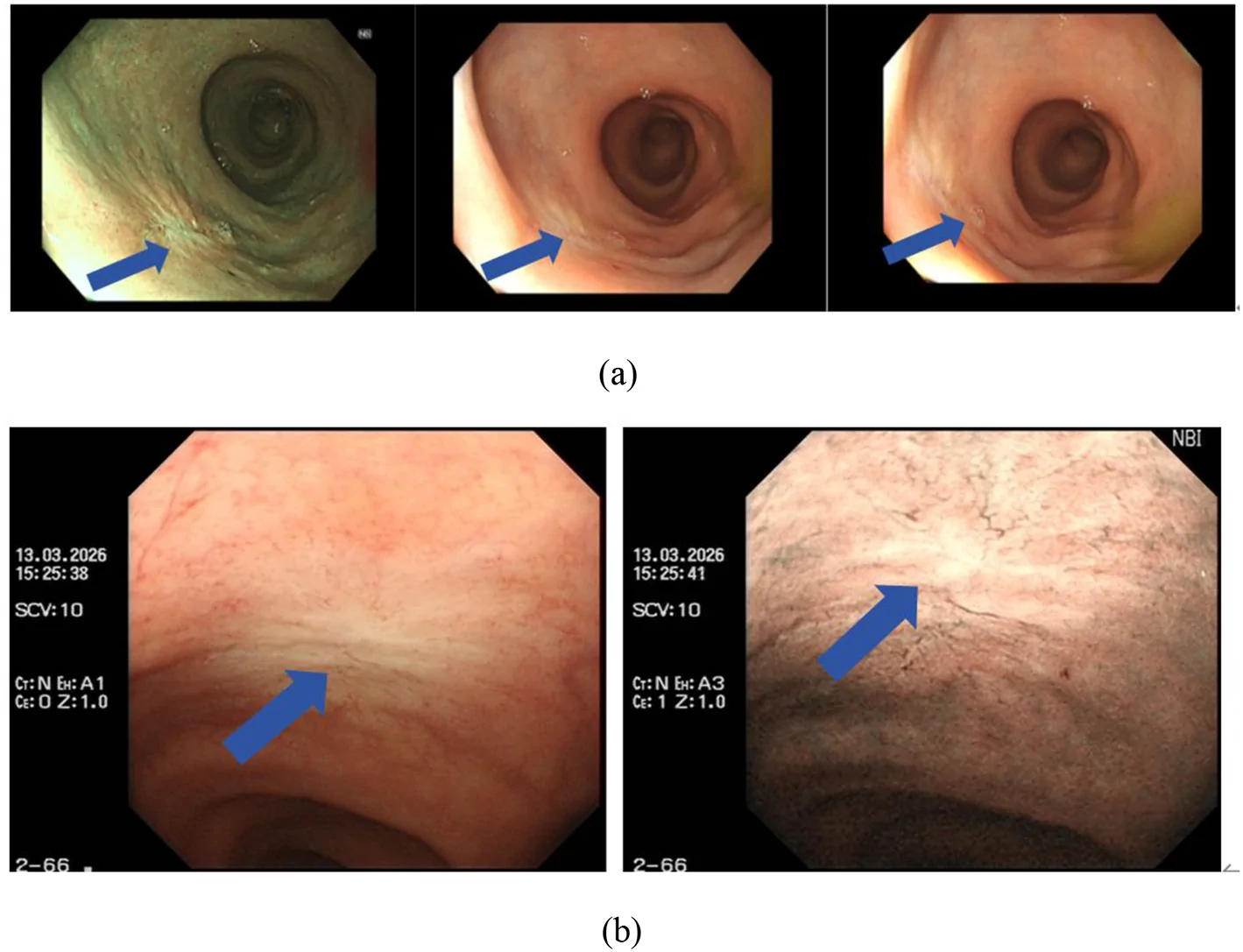
Colonoscopic findings during follow-up. **(A)** Colonoscopy on April 2025. The blue arrows indicate the linear white cicatricial contracture in the rectum; **(B)** Colonoscopy on March 2026. The blue arrows indicate the white flat scar.

Serial imaging on July 13 (CT), July 16 (MRI) (**Figure 2C**), and October 2025 (CT/MRI) (**Figure 2D**) consistently showed sustained mrTRG 1, T stage: T0 (no obvious mass at the original lesion site, no significant high signal on DWI sequence). Stable rectal wall thickening, essentially unchanged small mesorectal lymph nodes, and no new lesions.

At the final assessment on March 2026, CT revealed largely unchanged wall thickening, while the previously noted small lymph nodes were no longer definitively visualized. MRI showed localized T2 hypointensity and mucosal irregularity, consistent with post treatment changes (mrTRG 1, no mucin component increase, no significant high signal on DWI sequence) (**Figure 2E**). Colonoscopy identified a sTable 1.5 × 1.5 cm white flat scar on the right wall of the rectum, approximately 3 cm from the anal verge, with well defined borders and a smooth surface; under NBI, the capillary network was dilated and glandular openings were absent, confirming stability compared with the April 2025 findings (**Figure 3B**). Pathological biopsy of the scar confirmed chronic inflammation of the mucosa (**Figure 1C**). The final follow up diagnosis was clinical complete response (cCR) of rectal adenocarcinoma.

## 3 Safety

Grade 1–2 fatigue and diarrhea occurred during treatment, with no grade ≥3 immune-related adverse events (irAEs) reported. The patient had good tolerance and completed the full-course treatment.

## 4 Discussion

With advances in neoadjuvant immunotherapy research, molecular typing of colorectal cancer has become the cornerstone of individualized and precision therapy. Among molecular markers, mismatch repair status is one of the key indicators. Previous studies have demonstrated that immune checkpoint inhibitors (ICIs) bring transformative breakthroughs for patients with dMMR/MSI-H locally advanced rectal cancer (LARC), achieving high rates of clinical complete response (cCR) and pathological complete response (pCR) in a short period while significantly improving organ preservation success rates (11). In contrast, pMMR/MSS colorectal cancer exhibits low tumor mutational burden (TMB) due to genomic stability, failing to generate sufficient tumor neoantigens to activate naive T-cell responses and resulting in loss of immune recognition. This primary immune ignorance further evolves into a highly immunosuppressive microenvironment characterized by scarce infiltration and functional exhaustion of effector T cells, alongside massive accumulation of immunosuppressive cells including regulatory T cells (Tregs), myeloid-derived suppressor cells (MDSCs) and M2-type tumor-associated macrophages (TAMs), which secrete key inhibitory cy-tokines such as TGF-β and IL-10. Notably, abnormally activated TGF-β signaling not only directly suppresses T-cell function but also drives cancer-associated fibroblasts (CAFs) to form fibrotic barriers that physically block T-cell infiltration, leading to “immune privilege” (12). This multi-layered inhibitory mechanism collectively maintains the “cold” state of tumors, resulting in poor efficacy of prior single-agent immunotherapy.

In light of the “cold tumor” nature of pMMR/MSS colorectal cancer, current re-search strategies mainly focus on combining ICIs with anti-tumor agents via different pathways to enhance the efficacy of PD-1/PD-L1 inhibitors, thereby converting “cold tumors” to “hot tumors”. In studies of PD-1 monotherapy combined with short-course radiotherapy (SCRT) and chemotherapy, the TORCH trial administered SCRT followed by CAPOX plus Toripalimab for pMMR/MSS LARC, significantly increasing the cCR rate to 55.8%, with 80% of patients achieving sphincter preservation and manageable toxicity (13). The UNION trial, a randomized controlled multicenter study, used SCRT followed by CAPOX plus Camrelizumab and showed significant advantages in im-proving pCR rate, the pCR rates were 39.8% [95% confidence interval (CI) 30.7% to 49.5%] in the experimental arm compared to 15.3% (95% CI 9.3% to 23.0%) in the control arm (difference, 24.6%; odds ratio, 3.7; 95% CI 2.0-6.9; P < 0.001) (14). Regarding combined PD-1 and CTLA-4 blockade, a phase II randomized clinical trial (NCT02870920; registered Oct 12, 2016) (15) treated metastatic advanced pMMR/MSS colorectal cancer with Durvalumab plus Tremelimumab; the primary endpoint of overall survival (OS) showed a median OS extension of 6.6 months. Notably, subsequent subgroup analysis suggested that patients with a tumor mutational burden (TMB) ≥28 mut/Mb derived greater OS benefit from the combination regimen. The QL1706–208 study demonstrated that the combination of Iparomlimab and Tuvonralimab, Bevacizumab and XELOX achieved an objective response rate (ORR) of 70.6% in MSS metastatic colorectal cancer (mCRC), with 2 patients undergoing conversion resection (16).These findings confirm the application potential of immune combination therapy in pMMR locally advanced rectal cancer (**Table 2**).

**Table 2:** The ongoing trials on neoadj IO in pMMR LARC.

Trial	Registration	Phase	Population	Intervention	Key outcome
TORCH	NCT04586471	Phase II	pMMR/MSS LARC	Short-course RT → CAPOX + toripalimab	cCR rate: 55.8%; 80% sphincter preservation
UNION	NCT04928807	Phase III	pMMR/MSS LARC	Short-course RT → camrelizumab + CAPOX	pCR rate: 39.8% vs. 15.3% (LCRT control)
CO.26 (NCT02870920)	NCT02870920	Phase II	Metastatic pMMR/MSS CRC	Durvalumab + tremelimumab	mOS extension: 6.6 months; TMB ≥28 mut/Mb subgroup benefit
QL1706-208	NCT05329025	Phase I/Ib–II	MSS metastatic CRC	Iparomlimab + tuvonralimab + bevacizumab + XELOX	ORR: 70.6%; 2 conversion resections

In this case, a patient with pMMR/MSS rectal cancer achieved cCR evidenced by MRI within 12 weeks after neoadjuvant therapy with Iparomlimab and Tuvonralimab combined with 3 cycles of XELOX chemotherapy and 25 fractions of radiotherapy (45 Gy/25F) (4 cycles of Iparomlimab and Tuvonralimab in total). This immune combination strategy not only drastically shortened the time to cCR but also enabled individualized treatment and organ preservation through shared decision-making between physicians and patients, reducing cumulative toxicities from radiotherapy, multiple cycles of chemotherapy and surgery while improving quality of life. This outcome highlights the significant potential of immune combination therapy in enhancing treatment efficacy for MSS rectal cancer patients.

It is important to note that the standard neoadjuvant regimen for pMMR LARC in current guidelines consists of long-course capecitabine based chemoradiotherapy or short-course radiotherapy followed by consolidation chemotherapy. The addition of oxaliplatin to concurrent chemoradiotherapy has been investigated in trials such as STAR-01 and ACCORD-12, with no consistent demonstration of improved pCR or survival benefit but increased toxicity. This intensified regimen was selected by multidisciplinary team (MDT) consensus, considering the patient’s relatively young age, good performance status (ECOG-PS 1), and the patient had a pronounced desire for maximal tumor downstaging to optimize the probability of sphincter preservation.

The observed clinical response occurred in the context of multimodal therapy incorporating dual immune checkpoint blockade, radiotherapy, and chemotherapy. Preclinical studies have suggested that radiotherapy may promote antigen release and upregulate PD-L1 expression, that oxaliplatin may enhance immunogenic cell death, and that dual PD-1/CTLA-4 blockade may potentiate anti-tumor immunity through complementary mechanisms (10, 17–21). However, a single-case report is inherently unable to elucidate the underlying mechanisms of treatment response or to determine the specific contribution of each therapeutic modality. The discussion above is therefore confined to an objective description of the observed clinical phenomena, with no inference of definitive mechanistic conclusions.

Regarding safety, no grade ≥3 immune-related adverse events were observed. The favorable tolerability may reflect multiple factors, including the structural design of the MabPair^®^ platform (fixed 2:1 PD-1/CTLA-4 ratio), the short duration of immunotherapy exposure (4 cycles during induction, discontinued after cCR confirmation), and individual patient variability. Caution is warranted in attributing the safety profile solely to drug design, as the limited treatment duration and single-case nature preclude definitive conclusions.

This case reports a patient with pMMR LARC who achieved cCR following multimodal therapy incorporating Iparomlimab and Tuvonralimab. It is critical to emphasize that intensive chemoradiotherapy followed by consolidation chemotherapy alone can achieve cCR and enable non-operative management in more than 30% of patients with LARC. Based on this single observation, it is not possible to attribute the cCR to the addition of immunotherapy or to determine the specific contribution of any single modality. The role of Iparomlimab and Tuvonralimab in this setting remains investigational, and these findings should be interpreted as strictly hypothesis-generating. Immune combination therapy also faces practical challenges, including the risk of immune-related adverse events requiring close monitoring, and the complexity and cost of combination regimens.

This report has several inherent limitations. Firstly, as a single case observation, the findings lack generalizability and are subject to patient selection bias. Secondly, although we postulate a triple synergistic mechanism (radiotherapy, dual ICIs, and chemotherapy), the specific contribution of each modality to the observed cCR could not be delineated without paired biopsy sequencing data. Thirdly, the optimal duration of immunotherapy and the long-term durability of response beyond one year remain unknown. Therefore, our results should be interpreted as hypothesis generating, and prospective randomized clinical trials are urgently needed to validate this approach.

## 5 Conclusion

This report describes a case of MSS/pMMR rectal cancer that achieved cCR within a short period following multimodal neoadjuvant therapy incorporating Iparomlimab and Tuvonralimab, with a well-tolerated safety profile, and during the follow-up period of another year, no recurrence was observed. This outcome provides novel insights and approaches for MSS rectal cancer treatment. Future research should further explore biomarkers predictive of benefit from combination immunotherapy in pMMR colorectal cancer, and optimize radiotherapy dosage, fractionation schedules and timing of immunotherapy administration to achieve precision therapy and improve patient survival and quality of life. However, intensive chemoradiotherapy with consolidation chemotherapy alone can achieve cCR in more than 30% of patients with LARC. The specific contribution of immunotherapy to the observed response cannot be determined from a single case. These findings are hypothesis-generating and warrant validation in prospective clinical trials.

## Patient perspective

The patient expressed satisfaction with the non-surgical management and reported a significant improvement in his quality of life compared to the initial diagnosis. He is grateful for the opportunity to avoid surgery and a permanent colostomy.

## Data Availability

The original contributions presented in the study are included in the article/supplementary material. Further inquiries can be directed to the corresponding author.
